# Supplementary material for: Cardiopulmonary Injury in the Syrian Hamster Model of COVID-19
Source: Viruses. 2022 Jun 27;14(7):1403. doi: 10.3390/v14071403 (PMC9316644; doi:10.3390/v14071403)
Supplement: Supplementary file 1 [file viruses-14-01403-s001.zip › viruses-1730066-supplementary.pdf]

Supplemental Figures

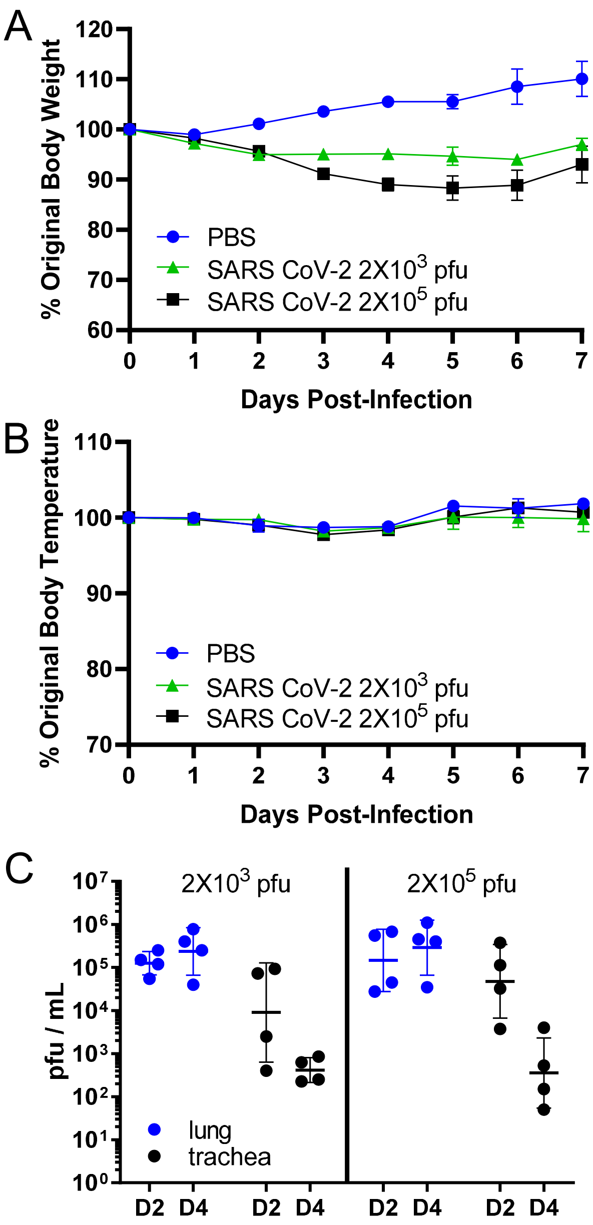

Figure S1. Weight and viral load in hamsters following intranasal infection with SARS-CoV-2 at a low and high dose. The weights (**A**) and temperature (**B**) of each male hamster (n=2 PBS group, n=4 infected groups) were measured daily from day 0 through day 7. Data are presented as mean ± SEM. (**C**) A plaque assay was used to measure the amount of infectious virus in whole lung or trachea at 2 and 4 dpi. Data are presented as geometric mean ± geometric SD.

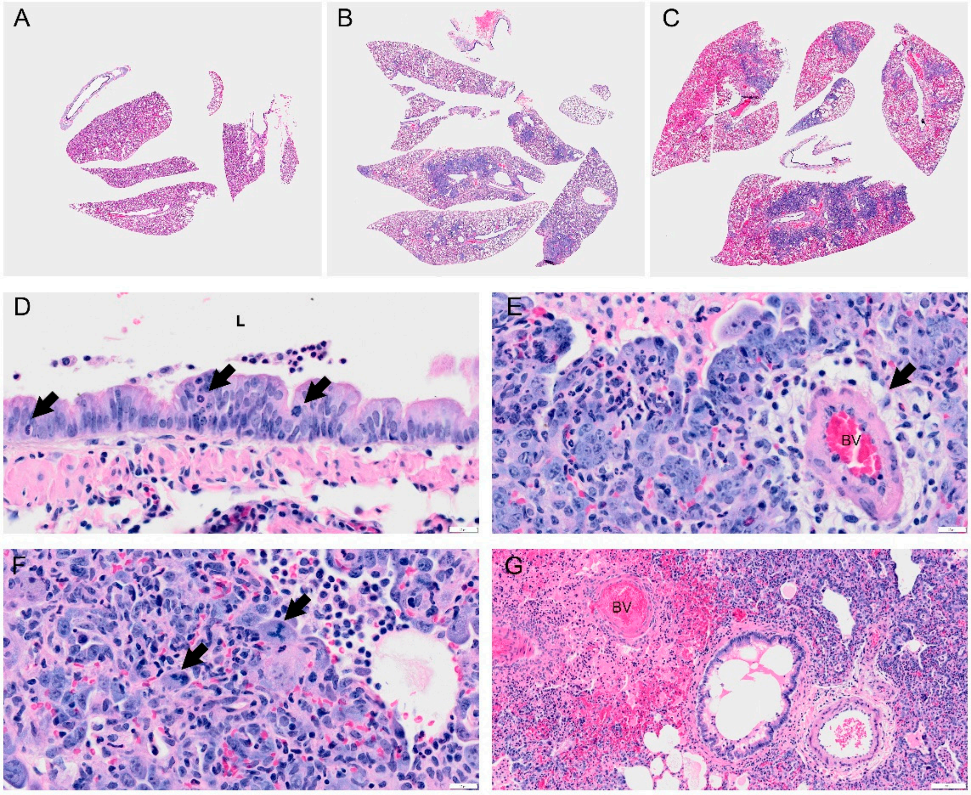

Figure S2. **Representative images of male Syrian hamster lungs from mock or SARS-CoV-2 infection**. (**A-C**) Images of whole lungs of hamsters at seven days post-infection following mock-infection (left), 2 X 10^3 PFU SARS CoV-2 WA1 (center), or 2 X 10^5 PFU SARS CoV-2 WA1 (right). Lungs were stained with hematoxylin and eosin. (**D**) Bronchial epithelium shows cell debris of macrophages, polymorphonuclear leukocytes, and necrotic epithelial cells in the bronchial lumen (L), and bronchial epithelial cell hyperplasia with mitoses (arrowheads) in the epithelial layer. Monocyte infiltration is prevalent in submucosa layer. (**E**) Representative image showing consolidation with perivascular edema and inflammatory cells (monocytes and polymorphonuclear cells, and fibroblasts) infiltration around a small artery (BV), eosinophilic exudate, neutrophils and eosinophils in alveolar air spaces, atypical hypertrophic epithelial cells with large nuclei and multinucleated cells. (**F**) Abundant mitosis and atypical mitosis of hyperplastic type II pneumocytes (arrowheads), macrophages and lymphocytes in alveolar air space. (**G**) In lungs infected at the high dose show fibrin aggregates in pulmonary blood vessels, with necrosis and hemorrhage in surrounding areas. A-C, Bar=2 mm; D-G, Bar=20 μm. Abbreviations: BV-blood vessel, L-lumen.

Table S1. Microscopic Pathology Scores for Syrian Hamsters at 1, 2-, 3-, 4-, and 8-Days Post-infection.

SEX	GROUP	DPI	Tracheal/bronchiolar necrosis/apoptosis/sloughing	Bronchial/bronchiolar exudates	Bronchiolar hyperplasia	Bronchiolar goblet cell metaplasia	Endothelial hypertrophy/margination	Peribronchiolar/perivascular inflammation	Interstitial inflam/septal thickening	Alveolar inflammation	Alveolar edema/hemorrhage	Extent of alveolar involvement	Consolidation	Bronchiolization
F	V	1	1	15	25	0	8	15	1	0	0	1	0	0
F	V	1	1	15	25	0	15	15	1	1	0	1	0	0
M	V	1	0	0	0	0	8	8	0	0	0	0	0	0
M	V	1	0	0	0	0	1	0	0	0	0	0	0	0
F	M	1	0	0	0	0	1	0	0	0	0	0	0	0
F	M	1	0	0	0	0	1	1	1	15	0	8	1	0
M	M	1	0	0	0	0	0	0	0	0	0	0	0	0
M	M	1	0	0	0	0	0	0	0	0	0	0	0	0
F	V	2	40	25	8	0	60	40	40	8	0	40	1	0
F	V	2	40	25	15	0	40	40	8	1	0	1	0	0
M	V	2	25	40	15	0	25	25	15	8	0	15	8	0
M	V	2	0	0	0	0	0	0	0	0	0	0	0	0
F	M	2	0	0	25	0	0	0	0	0	0	0	0	0
F	M	2	0	0	8	0	0	0	0	0	0	0	0	0
M	M	2	0	0	0	0	0	0	0	0	0	0	0	0
M	M	2	0	0	0	0	0	0	0	0	0	0	0	0

F	V	3	1	1	25	1	40	25	40	40	15	25	25	0
F	V	3	1	1	25	1	40	25	40	25	15	25	25	0
M	V	3	1	40	60	0	40	40	40	40	25	40	40	0
M	V	3	1	25	60	0	60	40	40	25	15	40	25	0
F	M	3	0	0	0	0	0	0	0	0	0	0	0	0
F	M	3	0	0	0	0	0	0	0	0	0	0	0	0
M	M	3	0	0	0	0	0	0	0	0	0	0	0	0
M	M	3	0	0	15	0	0	0	0	0	0	0	0	0
F	V	4	1	25	60	1	40	25	25	15	15	15	8	0
F	V	4	1	15	80	15	40	40	60	40	60	80	80	0
M	V	4	1	25	60	1	60	40	60	40	40	60	60	0
M	V	4	1	40	60	1	40	40	40	25	15	40	25	0
F	M	4	0	0	0	0	0	0	0	0	0	0	0	0
F	M	4	0	0	0	0	0	0	0	0	0	0	0	0
M	M	4	0	0	0	0	0	0	0	0	0	0	0	0
M	M	4	0	0	0	0	0	0	0	0	0	0	0	0
F	V	8	0	0	60	1	1	15	40	8	0	60	60	80
F	V	8	0	0	40	0	1	25	25	1	0	25	25	40
M	V	8	0	1	25	8	8	25	25	8	0	25	15	60
M	V	8	0	0	40	8	15	25	40	1	0	40	25	80
F	M	8	0	0	0	0	0	0	0	0	0	0	0	0

**F**	**M**	8	**0**	**0**	**0**	**0**	**0**	**0**	**0**	**0**	**0**	**0**	**0**	**0**
**M**	**M**	8	**0**	**0**	**0**	**0**	**0**	**0**	**0**	**0**	**0**	**0**	**0**	**0**
**M**	**M**	8	**0**	**0**	**0**	**0**	**0**	**0**	**0**	**0**	**0**	**0**	**0**	**0**

Abbreviations: Sex: M-male, F-female, Group: M- Mock-infected with PBS or S-SARS-CoV-2 infected; DPI-days-post-infection. Semiquantitative scores used 0-1 = within normal limits, 2-14 = Minimal: Rare or inconspicuous lesions; 15-39 = Mild: Multifocal or small, focal, or widely separated, but conspicuous lesions; 40-79 = Moderate: Multifocal, prominent lesions; 80-99 = Marked: Extensive to coalescing lesions or areas of inflammation with some loss of structure and 100 = Severe: Diffuse lesion with effacement of normal structure.

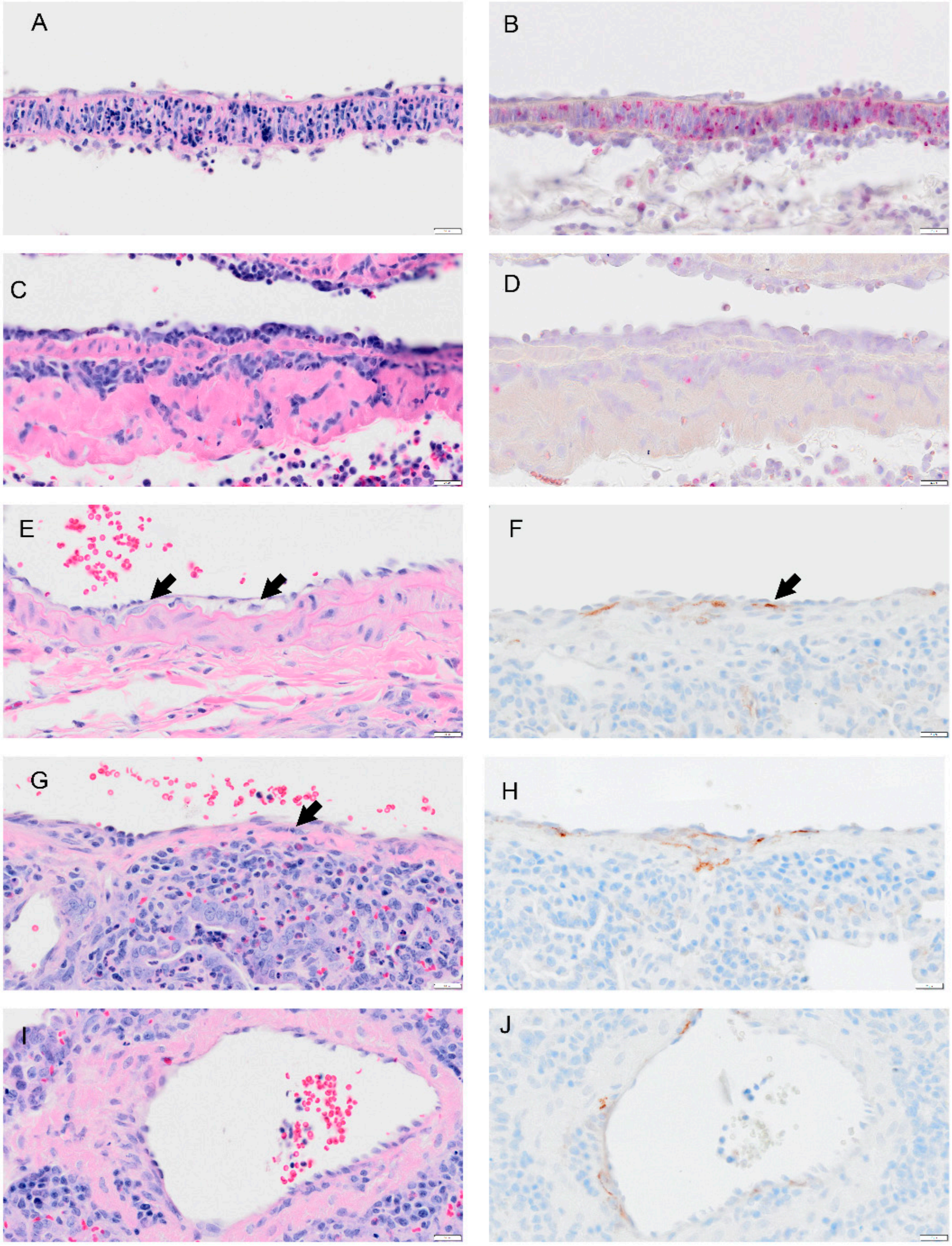
A
B
C
D
E
F
G
H
I
J

Figure S3. Additional pathology and immunohistochemistry of the large pulmonary blood vessels from Syrian hamster following infection with SARS-CoV-2. (**A**) H&E image of main pulmonary artery showing abundant neutrophils in tunica media of male hamster at 3 dpi. (**B**) Adjacent section to (A) with NACE stain. (**C**) H&E image of main pulmonary vein of a male hamster at 3 dpi showing clustered cells in tunica media. (**D**) Adjacent section to (C) with NACE stain. Stain shows most of the proliferated cells were not neutrophils. (**E**) H&E image of some cells in the main pulmonary artery located between basal membrane and endothelial cells (arrow) in female hamster at 8 dpi. (**F**) Adjacent section to (E) with ACE2 stain. Image shows ACE2 positive cells (arrow), in a female hamster at 8 dpi. (**G**) H&E image of main pulmonary vein of a female hamster on 8 dpi. Arrow shows cells in tunica intima. (**H**) ACE2 IHC of adjacent section of (G) which shows upregulated ACE2 signal with pericyte. (**I**) H&E image showing proliferation of cells in blood vessel wall in a female hamster on 8 dpi. A small pulmonary vein with spindle shape near basal membrane (pericytes), some round shape in tunica media (smooth muscle). (**J**) ACE2 IHC of adjacent section of (I) showing ACE2 upregulated in pericytes near basal membrane. Scalebar=20 µm

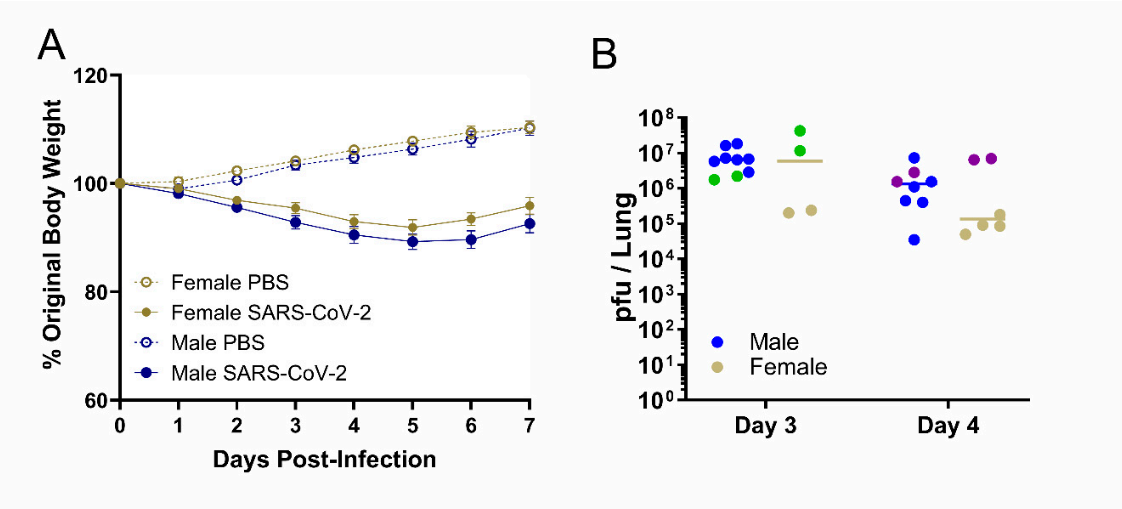

Figure S4. Representative weight and viral load in hamsters intranasally infected with SARS-CoV-2 at 2 x 10^5 pfu. In (A), we present body weight change for male hamsters (PBS n=5; SARS-CoV-2 n=8) and female hamsters (PBS n=6; SARS-CoV-2 n=8). Similarly, we present viral load in (B) which shows plaque assay titers in lungs of male and female hamsters at 3 dpi (male n=9; female n= 4) and 4 dpi (male n =8; female n=6). Green dots represent males and females at 3 dpi that have been shown in Figure 1; Purple dots represent males and females at 4 dpi that have been shown in Figure 1.
